# Transcriptional fingerprinting of regulatory T cells: ensuring quality in cell therapy applications

**DOI:** 10.3389/fimmu.2025.1602172

**Published:** 2025-06-16

**Authors:** Zhang Cheng, Li-Jie Wang, Yuchi Honaker, Steven A. Cincotta, Claire E. Page, Sydney Vollhardt, Victor Yuan, S. Alice Long, Yuanyuan Xiao, Joshua N. Beilke, Joseph R. Arron, Jeffrey A. Bluestone

**Affiliations:** ^1^ Sonoma Biotherapeutics, South San Francisco, CA & Seattle, WA, United States; ^2^ Center for Translational Immunology, Benaroya Research Institute, Seattle, WA, United States

**Keywords:** cell therapy, Treg product, Treg fingerprint, Treg identity, autoimmunity

## Abstract

**Introduction:**

The success of regulatory T cell (Treg) therapies depends on the source of Treg and the quality of the Treg manufacturing product that maintains Treg identity. Commonly used methods to identify Treg, including assessment of FOXP3 expression and demethylation of the Treg-specific demethylated region (TSDR), may not be sufficient on their own to ensure that Treg cell therapy drug products have an optimal identity and phenotype prior to infusion into patients.

**Methods:**

To address this critical need, we developed a robust framework to molecularly characterize Treg products using next-generation sequencing. By systematically profiling Treg and effector T cells (Teff) pre- and post-expansion, we defined the molecular fingerprints for expanded Treg products. We employed a non-parametric algorithm to score Treg manufacturing products for their cell identity and expansion fingerprints.

**Results:**

The identity fingerprint reflects Treg cell identity by effectively distinguishing Treg from Teff cells irrespective of their activation status, with 100% sensitivity and specificity, while the expansion fingerprint discriminates expanded versus endogenous Treg or Teff cells. We also showed that the identity fingerprint predicts Treg stability in in vitro settings and can be used to illustrate differences in drug products generated using distinct strategies. We further applied fingerprinting to bulk RNA sequencing (RNA-seq) data from endogenous and expanded Treg cells in a Phase 2 clinical trial for type 1 diabetes (T1D), demonstrating its ability to capture Treg identity and expansion in an independent study.

**Discussion:**

This Treg fingerprinting method provides a powerful tool to molecularly characterize Treg products, potentially enabling correlative analysis with the safety and efficacy outcomes of Treg-based cell therapies.

## 1 Introduction

The use of regulatory T cell (Treg) therapies to restore immune homeostasis and self-tolerance is a promising new modality for the treatment of autoimmune disorders. Early clinical trials in conditions such as type 1 diabetes (T1D) (1), multiple sclerosis (MS) (2), and inflammatory bowel disease (IBD) (3, 4) have demonstrated that these therapies are safe and well-tolerated and have shown promise in controlling autoimmune inflammation. Most Treg cell therapies developed thus far are autologous and require drug products to be manufactured for each individual patient. To ensure the highest standards, the identity, potency, quality, and purity of each drug product must be assessed, posing challenges for autologous cell therapies where these parameters require consistency across individual lots for each patient treated. Current release assays are focused on assessment of the phenotype and function of the final drug product and ensuring that these metrics align with endogenous Treg, however these methods alone may not reliably identify and validate the functional integrity of expanded Treg cells (3, 5). Accurately assessing the function of Treg cells also poses additional challenges in that Treg can suppress immune responses through several mechanisms (immunosuppressive cytokines, metabolic disruption, inhibitory coreceptors) that may not all be operational in all tissues or disease settings (6). Current potency assays cannot measure multiple mechanisms simultaneously, requiring individual assays to be developed and validated for use to capture the full suppressive potential of Treg cell therapies. In addition to these methods, a molecular profiling approach might identify specific gene or protein expression profiles that capture multiple aspects of an optimal Treg cell therapy drug product, allowing for an additional measure that can help to ensure these treatments meet therapeutic standards.

RNA sequencing (RNA-seq) allows for the measurement of the average gene expression across a population of cells (bulk RNA-seq) or at the individual cell level (single-cell [sc]RNA-seq). These technologies, along with computational methods such as machine learning algorithms and pathway-based tools like Gene Set Enrichment Analysis (GSEA) (7), single-sample GSEA (ssGSEA) (8), and gene set variation analysis (GSVA) (9), have enabled the identification and quantification of gene expression signatures (or “fingerprints”) that can help to define immune cell subsets. These analyses have become an essential tool in drug development and disease treatment. Fingerprinting has been used in applications ranging from drug discovery (10) to driving new insights into disease mechanisms and enabling precision medicine approaches to tailor treatments specifically to individual patients (11). In cellular therapies, gene expression analysis has been employed to identify gene signatures linked to successful and poor treatment outcomes (12, 13), provide insights into treatment resistance mechanisms (14), and examine interactions between CAR T cells and tumor microenvironments (15). These examples demonstrate the practical utility of this approach and further highlight the need to develop fingerprints for products currently in development.

Although well-established Treg markers such as FOXP3 and CD25 (IL2RA) have been identified, they are insufficient to reliably identify Treg as single markers due to overlapping, albeit transient, up-regulation in activated Teff. Demethylation of the Treg-specific demethylated region (TSDR), which is linked to stable FOXP3 expression (16), can also be used to identify Treg; however, this method may not capture the functional status of these cells (17). Advances in transcriptomics now offer a high-resolution view of gene expression, and several Treg transcriptional fingerprints have been published in the literature (18–20). These studies identified mRNA transcripts critical to Treg identity, including FOXP3, IL2RA, CTLA4, and TNFRSF18 (GITR), and other transcripts such as ENTPD1 (CD39), TGFB1, and LRRC32 (GARP), which have been identified as markers of Treg suppressive function. Of these 3 studies, however, only one (Pesenacker, et al.) considered the activation status of Treg in the analysis, generating an activation-independent Treg identity fingerprint (19). In the context of Treg cell therapies that typically undergo multiple rounds of activation and expansion prior to infusion into patients, an activation-independent Treg identity fingerprint would be required to remove genes upregulated upon activation from the gene signature that defines cell identity. This is especially true given the overlap in gene expression between Treg and activated Teff cells.

Like most other immune cells, Tregs demonstrate some level of plasticity in their phenotype under certain conditions, (21) including T helper (Th)-like subsets based on expression of genes observed in effector Th subset counterparts (22). In addition, in many autoimmune diseases, these Th-like Treg subsets may lose suppressive function, which may ultimately contribute to autoimmune inflammation (23–25). Thus, it is essential that Treg molecular fingerprints take advantage of canonical Treg gene expression transcripts that identify effector functions. To address this, it is critical to identify genes that are uniquely expressed in expanded Treg and Teff and to incorporate this information into a molecular fingerprint that provides a more comprehensive view of the final drug product.

Sonoma Biotherapeutics (SBT) is developing an autologous Treg cell therapy engineered with a chimeric antigen receptor (CAR) to respond to activation signals in inflamed tissues. Using both internal and published data, we developed two Treg fingerprints that can be used to assess final drug product: a Treg identity fingerprint that can differentiate between Treg and Teff cells regardless of their expansion state, and a Treg fingerprint to characterize expanded Treg after the manufacturing process. Both were validated using published and internal data and were applied to both nonclinical and clinical datasets to demonstrate practical applications of the fingerprints. Together, these data support the development and use of Treg fingerprints as part of the drug development process, not only for use in QC of the final drug product, but also to inform the design of clinical trials.

## 2 Materials and methods

### 2.1 Generation of Treg and Teff cells

Treg (CD4^+^CD25^hi^CD127^lo^) and Teff (CD4^+^CD25^lo^CD127^hi^) were isolated from peripheral blood mononuclear cells (PBMC) by fluorescence-activated cell sorting (FACS). PBMCs were collected by Ficoll (GE Healthcare; Cat#17-1440-03) based precipitation by 1000g centrifuge at room temperature for 20 minutes, then PBMCs were processed by anti-CD25 isolation beads (Miltenyi; Cat#170-076-717) to enrich CD25^+^ cells. Enriched CD25^+^ cells were then stained by anti-CD4/CD25/CD127 before Treg and Teff isolation by FACS (Sony MA900). Purified cells were then either frozen (D0) or activated and expanded for 14 days in CTS™ OpTmizer™ T Cell Expansion media (ThermoFisher Scientific) with anti-CD3/anti-CD28 Dynabeads (ThermoFisher Scientific) (D14). Treg were transduced with 2^nd^ generation CAR constructs (scFv-CD28z) on day 3.

### 2.2 Treg suppression assays

CD3^+^ responder T cells (Tresp) were isolated from cryopreserved PBMC (StemCell Technologies; Cat# 19051) and labeled with CellTrace™ CFSE (ThermoFisher; Cat# C34554) before plating 5×10^4^ cells/well in a 96-well plate. D14 Treg, destabilized Treg, or 4 stim Teff cells were plated (>95% cell viability determined by NucleoCounter NC-202 cell counter) for a 2-fold dilution series of the test sample:Tresp ratios (1:1, 1:2, 1:4, 1:8, 1:16, 1:32, 1:64 and 1:128), and rested overnight in complete RPMI 1640 (cRPMI) + 300 IU/mL IL-2. Cocultures and Tresp alone were cultured in cRPMI media + anti-CD3/anti-CD28 Dynabeads for 3 days. All suppression assays were executed in duplicate with 5 different donors, and CD4^+^ and CD8^+^ Tresp cell proliferation was measured by CFSE-dilution measured by flow cytometry. The % suppression was calculated by the following equation, then the mean value and standard error of the mean were calculated from the data of the 5 donors:


$$100\%\times \frac{\left(\text{\% CFSE dilution of Tresp only-\% CFSE dilution of Tresp from coculture}\right)}{\text{\% CFSE dilution of Tresp only}}$$

### 2.3 RNA-seq experiments

RNA-seq experiments were conducted to investigate transcriptomic changes under various conditions. Cell pellets (1×10^6^ cells) were shipped to an external vendor (SeqMatic, LLC, Fremont, CA), who performed sample preparation, RNA extraction, RNA QC, Illumina Standard mRNA library preparation (input concentration following the recommend range 25–1000 ng) and sequencing by NovaSeq X Plus (at least 25M reads per sample with a paired-end read length of 150bp). Raw FASTQ files were then provided by the vendor. Samples with an RNA integrity (RIN) score below 8 did not proceed to library preparation.

#### 2.3.1 Treg transcriptional profiling post-thaw and after 24 hour culture

Cryopreserved D14 Treg cells generated from 6 donors were thawed and harvested for transcriptional analysis either immediately after thaw, or after 24 hour culture in cRPMI medium with 10% FBS and 300 IU/mL IL-2.

#### 2.3.2 Transcriptional profiling on Tregs and Teffs before/after destabilization

Cryopreserved D14 Treg cells expressing a high tonic signaling CAR (high-affinity scFv specific for myelin oligodendrocyte glycoprotein [MOG], fused to a CD28 costimulatory domain and CD3ζ signaling domain) and D14 Teff were thawed and cultured at 37°C for 24 hours in complete RPMI 1640 (cRPMI) medium + 10% FBS with 300 IU/mL IL-2. Cells were then plated at 5×10^5^ cells/mL in cRPMI with 300 IU/mL IL-2 and anti-CD3/anti-CD28 Dynabeads at a 2:1 cell to bead ratio. Beads were removed on Day 3, then cells were cultured for 4 days. The stimulation process was repeated 3 times, for a total of 4 rounds of stimulation over 28 days. Cells were collected immediately after thaw, and after 4 rounds of stimulation for transcriptional profiling. Unstimulated D0 and D14 Treg cells from the same donors were also analyzed.

### 2.4 RNA-seq data processing

#### 2.4.1 Internal datasets

FASTQ files for each dataset were processed using the nf-core/rnaseq pipeline (version 3.14.0) (26). The reference genome used was GRCh38 (Homo_sapiens.GRCh38.dna_sm.primary_assembly), and gene annotation was the Ensembl release 110. The pipeline performed the following steps: 1) Quality control of raw reads (FastQC), 2) Adapter trimming and quality filtering (Trim Galore), and 3) Transcript quantification (Salmon). The output of the gene-level transcript-per-million (TPM) matrix was used for downstream fingerprinting score calculation. The counts files were used for downstream differential gene expression analysis.

#### 2.4.2 External datasets

Three external datasets were included in the analysis: 1) Honaker et al., 2020 (data downloaded from doi:10.5061/dryad.02v6wwq08) (27), 2) GSE253540 (28), and 3) GSE243270 (29). The FASTQ files for these datasets were obtained using the nf-core/fetchngs pipeline and were processed following the same pipeline and parameters as the internal datasets to ensure consistency in data processing.

#### 2.4.3 Differential gene expression analysis

Differential gene expression analysis was performed using DESeq2 (30). Genes were considered differentially expressed if they met the following criteria: Log2 fold change > 1 and Banjamini-Hochberg adjusted p-value < 0.05.

### 2.5 Gene signature curation

#### 2.5.1 Treg identity signatures

**Figure 1:**
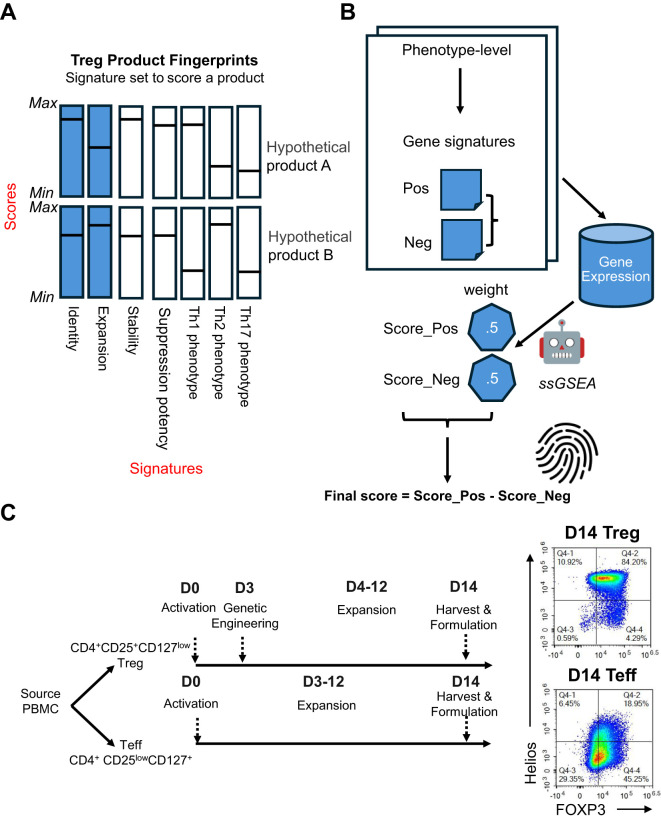
Overview of the SBT Treg product molecular fingerprints. **(A)** Molecular fingerprints are defined by 2 components: signatures underlying different Treg phenotypes, and metrics for scoring each signature. Signatures that have been developed and discussed here are illustrated by blue bars, with potential signatures for future development illustrated by white bars. Hypothetical score values for each signature are represented by a horizontal line. Together, these signatures and their respective scores could be used to differentiate cell therapy drug products, as demonstrated by hypothetical products A and B, shown here. **(B)** For each phenotype, a positive (Score_Pos) and negative (Score_Neg) score is derived using the ssGSEA approach based on predefined phenotype-specific gene sets, with each score carrying equal weight. The final score is computed by subtracting Score_Neg from Score_Pos. **(C)** Signatures were primarily derived using D0 (endogenous/unexpanded) Treg (CD4+CD25+CD127low) and Teff (CD4+CD25lowCD127+) cell sorted from PBMC, and D14 Treg and Teff that have undergone activation and expansion for 14 days. Representative flow cytometry plots demonstrate expression of FOXP3 and Helios in D14 Treg and Teff.

The Ferraro Treg identity signatures were obtained from Dataset S2 of Ferraro et al., 2014 (18) comprising 194 Treg-up and 192 Treg-down genes. The Pesenacker et al. signature was extracted from Figure 1F of their study (19).

To derive the SBT expansion-independent Treg identity signatures, we employed a two-step process: 1) Differential gene expression analysis between Treg and Teff was conducted at D0 and D14 separately. 2) Intersection of the gene lists was derived as: Treg identity signature genes—genes upregulated in both D0 and D14 Treg (compared to Teff) and Teff identity genes—genes upregulated in both D0 and D14 Teff (compared to Treg). Further, due to the availability of only one dataset containing both D0 Teff and Treg, we used the Treg/Teff signatures from Ferraro et al. (18) to perform the intersection. Two internal discovery datasets were included in the derivation of the Treg identity signature: SBT dataset 1 and SBT dataset 2 (**Supplementary Table S1**).

#### 2.5.2 Treg expansion signatures

Treg expansion signatures were derived by comparing D14 to D0 Tregs from two internal datasets: SBT dataset 2 and SBT dataset 3 (**Supplementary Table S1**). The final gene signatures (**Supplementary Table S2**) consisted of overlapping genes from these two datasets.

#### 2.5.3 STRING db protein-protein interaction map

Protein-protein interaction maps were generated using R Package “rbioapi:: rba_string_network_image”, with the following parameters: “required_score = 500” and “network_flavor = actions”.

### 2.6 SBT fingerprint score calculation

The SBT Fingerprint Score was calculated in two steps: 1) calculate the sub-scores for the positive (favorable) signature and the negative (unfavorable) signature separately and 2) subtract the negative sub-score from the positive sub-score. Calculation was performed using the R package GSVA with the following parameters: “method=ssgsea”, “diff.score=FALSE” for the input log2-transformed TPM expression matrix (per dataset).

## 3 Results

### 3.1 Overview of SBT Treg molecular fingerprints

Several statistical methods were studied for the development of the SBT Treg molecular fingerprint algorithms, including gene set variation analysis (GSVA) (9), single-sample gene set enrichment analysis (ssGSEA) (8), and singscore (31, 32). Ultimately, ssGSEA was chosen as the computational method to provide a sample specific summary of gene expression of the SBT Treg cell therapy product due to its precision, sensitivity, and robustness in analyzing single samples and its established use in similar applications (33).

The SBT Treg molecular fingerprint algorithm was defined by two components: gene signatures underlying different Treg phenotypes and metrics for scoring each signature (**Figure 1A**). Each fingerprint was developed using bidirectional gene signatures where each sample was scored on positive (“favorable”) and negative (“unfavorable”) gene signatures, or “sub-scores”. Positive and negative sub-scores were given equal weight, and the final score was calculated by subtracting the negative sub-score from the positive sub-score, enabling the ability to evaluate a cell product and determine whether the product exhibits the desired characteristics while avoiding unwanted characteristics (**Figure 1B**).

SBT Treg cell therapy drug product (D14 Treg) was generated from endogenous (D0) Treg (CD4^+^CD25^hi^CD127^lo^) isolated from peripheral blood mononuclear cells (PBMC) and activated, transduced with a CAR construct, then expanded for 14 days using anti-CD3/anti-CD28 beads [Materials & Methods, also see details from Stoops et al. (34)]. In these studies, we also included counterpart endogenous (D0) Teff (CD4^+^CD25^lo^CD127^hi^) and Teff cells that were activated and expanded similarly to Treg cells without genetic engineering (D14 Teff). All SBT-derived Treg samples used in this study (except those following destabilization experiments, described later) were good quality (**Supplementary Figure S1**). A comparison of the protein expression of the Treg markers FOXP3 and Helios between representative D14 Treg and D14 Teff samples demonstrated that Treg maintained high expression of both markers (typically ≥ 95% double positive) with significantly lower expression in D14 Teff (**Figure 1C**).

### 3.2 The Treg core identity fingerprint can accurately identify Treg cells irrespective of expansion state

**Figure 2:**
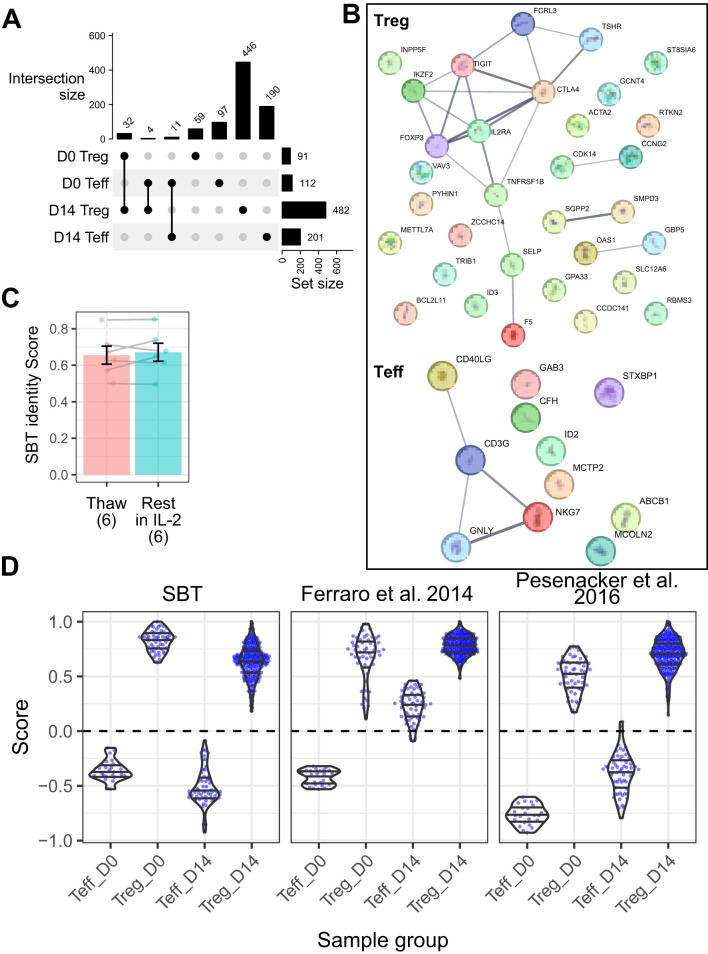
Sonoma Biotherapeutics (SBT) Treg identity signature distinguishes Treg from Teff in both resting and expanded states. **(A)** UpSet plot illustrating shared gene expression across 4 populations: Treg and Teff at D0 and D14. The matrix below represents intersections, with filled circles indicating which populations contribute to each intersection. The bar chart above the matrix shows the number of shared genes for each intersection, and the bar chart to the right displays the total number of expressed genes in each population, highlight the extent of overlap in gene expression across cell subsets and time points. The final Treg and Teff signatures were selected from the genes overlapping between D0 and D14. **(B)** Protein-protein interaction networks from StringDB for Treg signature genes (n=32, top) and Teff signature genes (n=11, bottom). Lines connecting genes represent functional and physical protein associations, with the line thickness indicating the strength of data support (minimum interaction confidence of 0.5). **(C)** Comparison of SBT Treg identity scores between D14 Treg samples (n=6) that were freshly thawed and the same samples that were rested for 24 hours in IL-2 (Paired t-test, P=0.44). **(D)** Comparison of scores derived from the SBT Treg identity fingerprint (left) with published fingerprints from Ferraro et al. (18) (middle) and Pesenacker et al. (19) (right) applied to D0 and D14 Teff and Treg generated by SBT. Each point represents an individual sample.

The SBT Treg identity fingerprint was developed using data from 3 studies: 2 internal bulk RNA-seq datasets (SBT dataset 1 and 2, **Supplementary Table S1**) containing D0 and D14 Treg (n=8 and n=45, respectively) and Teff (n=8 and n=21, respectively), and one public microarray dataset containing D0 Teff and Treg gene signatures from healthy donors (n=78), or donors with type 1 (n=60) or type 2 (n=30) diabetes (18). To identify fingerprints that could differentiate between Treg and Teff cells irrespective of expansion state, only genes differentially expressed in both D0 and D14 cells were considered. These analyses uncovered 32 genes in Treg and 11 genes in Teff that were differentially expressed at both time points and across different datasets (**Figure 2A**, **Supplementary Table S2**). The positive Treg identity gene signature contains genes typical of the Treg cell type, including FOXP3, IL2RA (CD25), IKZF2 (Helios), and CTLA4. The negative Treg identity gene signature (containing genes expressed in Teff), on the other hand, contained genes typically associated with Teff cells including CD40LG, and 2 genes (GNLY and NKG7) which are predominantly expressed in cytotoxic lymphocytes (**Figure 2B**). The reproducibility of the SBT Treg identity score was assessed by comparing scores of 6 D14 Treg samples immediately following thaw as well as following an overnight culture in the presence of IL-2, demonstrating that there were no significant differences in Treg identity scores between the 2 groups (**Figure 2C**).

We then compared the performance of the SBT Treg identity fingerprint to fingerprints based on other published signatures by applying each signature to a group of 12 internally derived and 1 externally derived validation datasets containing a total of 54 D0 Treg, 252 D14 Treg, 24 D0 Teff, and 59 D14 Teff samples (**Supplementary Table S1**). The SBT Treg identity fingerprint accurately differentiated between Teff and Treg at both D0 and D14, with Teff scores below 0 and Treg scores above, and little variation between experiments. The Treg fingerprint published by Ferraro, et al. (18) based on D0 Treg signatures (vs D0 Teff) accurately identified D0 Teff and D0 and D14 Treg, however D14 Teff scores were generally above 0 and not distinguished from Treg. A third fingerprint based on activation-independent Treg signatures published by Pesenacker, et al. (19) performed similarly to the SBT Treg identity fingerprint, differentiating cells at both time points (**Figure 2D**).

To determine whether the SBT Treg identity fingerprint enabled better resolution between cell types and time points than identifying Treg by expression of FOXP3 only, we analyzed D0 and D14 Treg and Teff gene expression of FOXP3. As expected, FOXP3 was highly expressed in D0 and D14 Treg, with only minimal expression in D0 Teff. D14 Teff, however, had moderate expression of FOXP3, which has been well described as being transiently upregulated in activated Teff cells (35, 36). Although FOXP3 gene expression in D14 Teff was lower than in Treg, it was still higher than in D0 Teff; in this case, by incorporating information from other relevant genes, the SBT Treg identity fingerprint provided better resolution between Treg and Teff, with Teff having similar scores below 0 irrespective of expansion (**Supplementary Figure S1**).

The accuracy, sensitivity, specificity, positive predictive value (PPV) and negative predictive value (NPV) of the SBT Treg identity fingerprint on the validation datasets were all 100%. The identity fingerprints described by Pesenacker et al. (19) also performed well with all metrics ≥98%, while the fingerprint described by Ferraro et al. (18) showed lower accuracy (86%), specificity (34%) and PPV (85%) than the other 2 fingerprints (**Supplementary Table S3**).

### 3.3 The Treg expansion fingerprint identifies T cells that have expanded, regardless of subset

The Treg identity fingerprint accurately distinguishes between Treg and Teff regardless of expansion, but it was not designed to assess whether the cells were properly expanded. To evaluate expansion, we developed a second fingerprint that is independent of cell identity and specifically distinguishes between unexpanded and expanded cells.

**Figure 3:**
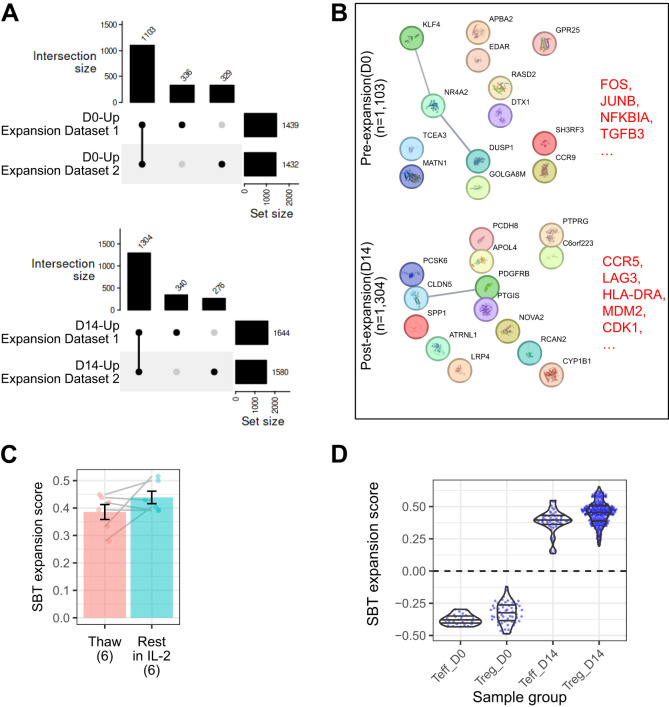
The SBT Treg expansion signature distinguishes between samples before and after expansion. **(A)** UpSet plot illustrating shared gene expression across D0 (top) and D14 (bottom) Treg. The matrix below represents intersections, with filled circles indicating which experiment contributes to each intersection. The bar chart above the matrix shows the number of shared genes for each intersection, and the bar chart to the right displays the total number of expressed genes in each experiment, illustrating the extent of overlap in gene expression across experimental replicates and time points. The final Treg expansion signature was selected from the genes overlapping between the 2 experiments. **(B)** Protein-protein interaction networks from StringDB for the D0 (top) and D14 (bottom) signatures. Lines connecting genes represent functional and physical protein associations, with the line thickness indicating the strength of data support (minimum interaction confidence of 0.5). **(C)** Comparison of SBT Treg expansion scores between D14 Treg samples (n=6) that were freshly thawed and the same samples that were rested for 24 hours in IL-2 (Paired t-test, P=0.18). **(D)** SBT Treg expansion scores applied to D0 and D14 Teff and Treg generated by SBT. Each point represents an individual sample.

Two internal datasets were used to generate the Treg expansion fingerprint, containing a total of 30 D0 and 48 D14 Treg samples. Genes that overlapped at each timepoint in each dataset were selected for the gene signature (**Figure 3A**). This analysis identified 1103 genes (**Supplementary Table S2**) differentially expressed in unexpanded D0 Treg (negative gene signature), including genes involved in regulating proliferation (FOS, JUNB, NFKBIA). 1304 genes were uniquely expressed in post-expansion Treg (positive gene signature) (**Supplementary Table S2**). These genes included the coinhibitory marker LAG3, CCR5, which allows for homing to sites where Teff are activated, HLA-DRA, linked to T cell activation, and CDK1 and MDM2, both of which drive proliferation (**Figure 3B**). The expansion fingerprint was applied to 6 D14 Treg samples immediately after thaw and again after overnight culture in the presence of IL-2. Among these donors, three demonstrated stable expansion scores and three showed increased scores after resting, however the difference was not statistically significant (**Figure 3C**, p-value = 0.18).

When applied to the group of 13 validation datasets, the SBT expansion fingerprint demonstrated 100% accuracy, sensitivity, specificity, PPV, and NPV. The SBT Treg expansion fingerprint accurately differentiated the expansion state of Treg with little inter-experimental variability, and despite being developed using only Treg gene signatures, the expansion fingerprint also accurately differentiated the expansion state of Teff (**Figure 3D**).

### 3.4 Using the SBT identity fingerprint to detect “destabilized” Treg

The applications of Treg fingerprinting can extend beyond confirming the identity and expansion of final drug product. For example, it has been observed that in some stressful conditions such as repetitive stimulation (37) or the expression of a high tonic signaling CAR that leads to a cell receiving chronic activation signals in the absence of antigen (38–40), FOXP3 expression in Treg can become unstable. Ultimately, these conditions can lead to a CD4^+^CD25^lo^FOXP3^lo^ population of destabilized Treg termed “exTregs” (41, 42). Although the precise mechanism of conversion of Treg to exTreg *in vivo* is somewhat controversial, they are hypothesized to have decreased regulatory function and may even adopt some effector characteristics (43).

**Figure 4:**
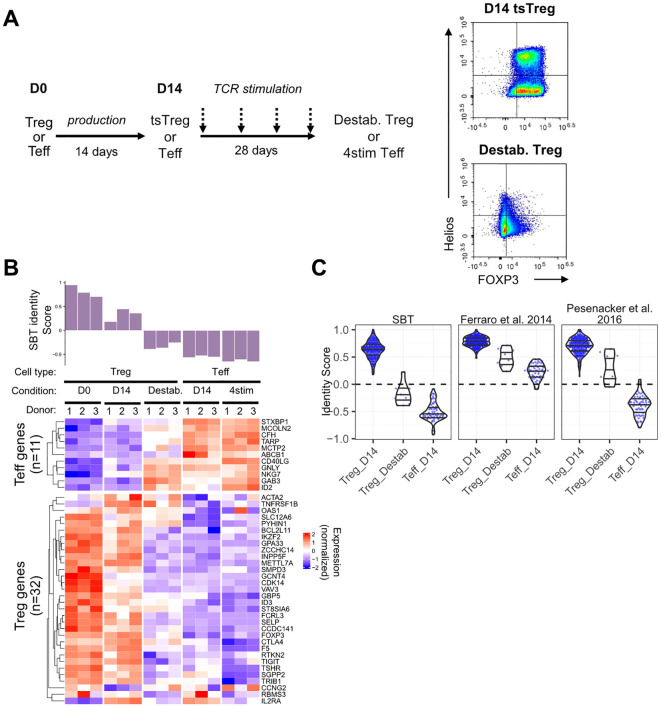
Application of the SBT Treg identity signature to identify destabilized Treg. **(A)** To generate destabilized Treg, Treg expressing a high tonic signaling CAR (D14 tsTreg) were stimulated using anti-CD3/anti-CD28 beads for 3 days and rested for 4 days before repeating for a total of 4 rounds of stimulation prior to transcriptional analysis. D14 Teff cells were also stimulated in the same manner as a control (4stim Teff). Representative flow plots demonstrate expression of FOXP3 and Helios in D14 tsTreg and destabilized Treg. **(B)** SBT Treg identity signature applied to Treg (D0, D14 tsTreg, and destabilized) and to Teff (D14 and 4stim) from 3 donors. Identity scores are shown in the bar chart (top) with normalized expression of the genes comprising the negative and positive Treg identity signature scores (bottom). **(C)** Comparison of scores derived from the SBT Treg identity fingerprint (left) with published fingerprints from Ferraro et al. (18) and Pesenacker et al. (19) when applied to D14 Treg, destabilized Treg, or D14 Teff generated by SBT. Each point represents an individual sample.

To assess the ability of the Treg identity fingerprint to differentiate between Treg and destabilized Treg, we employed an *in vitro* system wherein Tregs were transduced with a CAR construct containing a high-affinity scFv specific for myelin oligodendrocyte glycoprotein (MOG), fused to a CD28 costimulatory endodomain and CD3ζ. This construct exhibits high tonic signaling (data not shown) and was selected as a tool to induce destabilization. D14 Treg expressing the high tonic signaling CAR (D14 tsTreg) were repeatedly stimulated every 7 days through the TCR/CD28 for 28 days (4 times total), resulting in cells defined as destabilized Treg. These cells were compared to D14 Teff and D14 Teff that were stimulated in the same manner as destabilized Treg (4 stim D14 Teff). Compared to unstimulated D14 tsTreg, destabilized Treg had lower expression of FOXP3 and Helios as measured by flow cytometry (**Figure 4A**) and reduced suppressive function against both CD4 and CD8 T cells (**Supplementary Figures S2A, B**).

The SBT Treg identity scores of these populations quantitatively reflected the phenotype difference (**Figure 4B**). D0 Treg had the highest Treg identity scores, followed by D14 tsTreg which had reduced scores compared to D0 Treg due to the high tonic signaling activity of the CAR during the expansion protocol. Destabilized Treg had the lowest identity scores of the Treg included in this study with scores less than 0, due both to higher expression of Teff genes and lower expression of Treg genes, however, these scores were still higher than D14 Teff and 4 stim D14 Teff (**Figure 4B**). The gene expression profiles of the fingerprint genes match with the score evaluation showing a transition from stable D0 Treg to destabilized Treg to Teff. We further examined the relationship between Treg identity score and suppressive potential in 2 of the 5 donor samples for which matching identity scores and suppression assay data were available. The identity score significantly correlated with the maximum percent suppression of both CD4^+^ and CD8^+^ T cells at a 1:1 Treg: Tresp ratio. However, when suppression was quantified using the area under the curve (AUC) (44) across Treg: Tresp ratios from 1:1 to 1:128, the correlation did not reach the same statistical significance as with maximum percent suppression (**Supplementary Figure S2C**).

Subsequently, we compared the performance of the SBT Treg identity fingerprint with those based on Ferraro et al. (18) and Pesenacker et al. (19) by applying them to the gene expression data generated from this experiment. The three identity scores showed the same decreasing trend from D14 Treg to destabilized Treg and D14 Teff (**Figure 4C**). However, only the SBT Treg identity fingerprint assigned scores generally below 0 to destabilized Treg while the scores assigned by the 2 published identity fingerprints were generally above 0 and closer to scores of D14 Treg (**Figure 4C**).

### 3.5 Applying the SBT identity and expansion fingerprints to characterize different Treg drug products

Treg identity fingerprints might also be used to better understand differences between Treg drug products. Currently, there are multiple methods used to generate Treg cell therapies, including variations on the cell type used as the starting material. One alternative to isolating and expanding Treg from patients is to use CD4^+^ T cells as the starting material and to overexpress FOXP3, which has been shown to upregulate Treg associated genes such as CTLA4, IL2RA, and TNFRSF18, increase the production of suppressive cytokines IL-10 and TGF-β, and to enable the cells to exert some suppressive effects *in vitro* and *in vivo* (27). This method provides some advantage over the use of Treg which have smaller numbers in PBMC compared to CD4^+^ T cells and require extensive expansion to generate enough cells for infusion, however it is unknown whether forced expression of FOXP3 alone is sufficient to drive gene expression similar to Treg.

**Figure 5:**
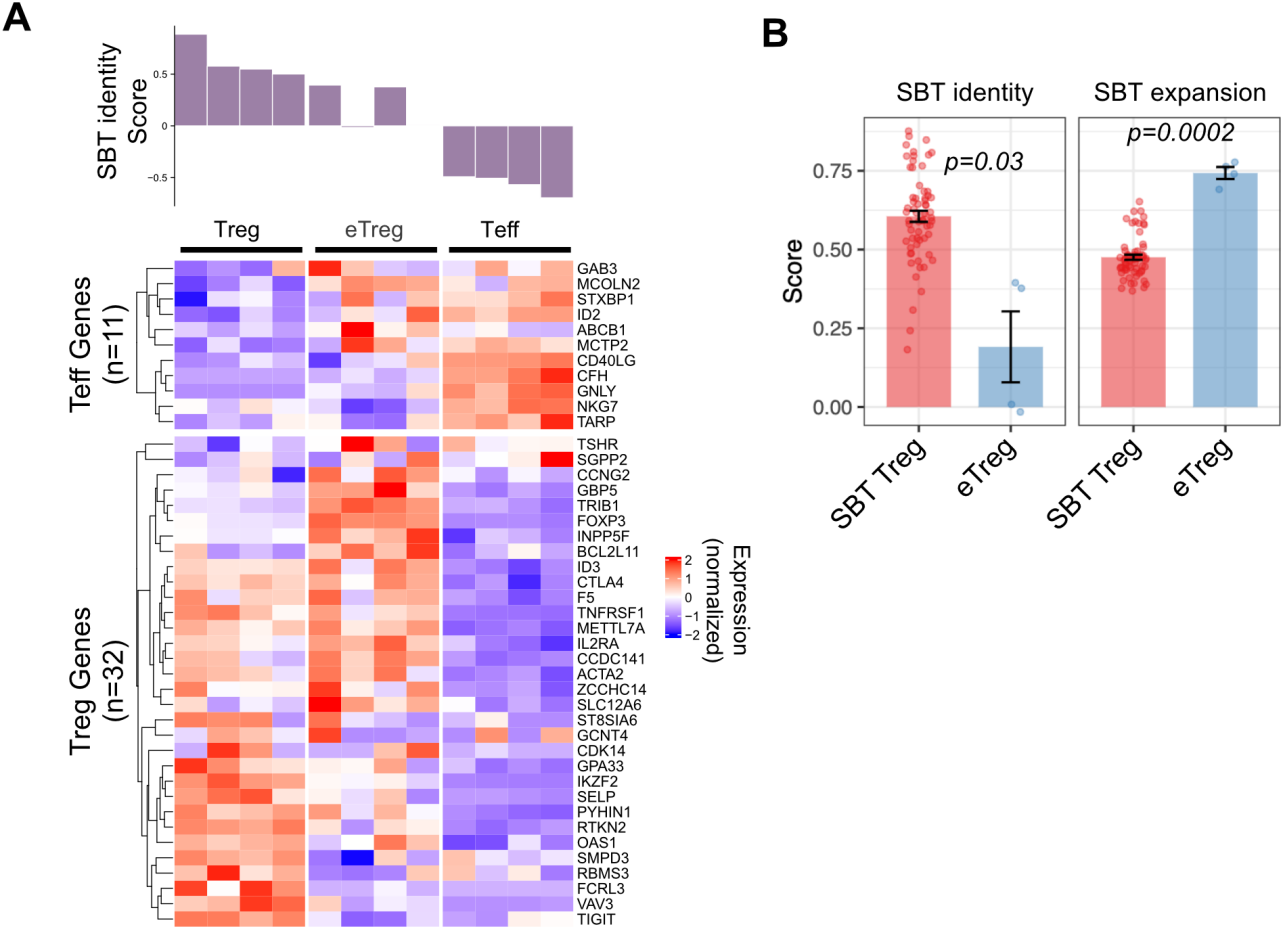
Application of SBT Treg signatures to differentiate Treg cell products. **(A)** SBT Treg identity score applied to activated Treg, ectopic FOXP3 CD4+ cells (eTreg) and activated Teff from data generated by Honaker, et al. (27). Identity scores are shown in the bar chart (top) with normalized expression of the genes comprising the negative and positive Treg identity signature scores (bottom). **(B)** Comparison of SBT Treg identity (left) and expansion (right) scores between D14 Treg derived by SBT and eTreg product. P-values calculated using Welch’s two-sample, two-sided t-test.

We applied the SBT Treg identity fingerprint to a published dataset that compared activated bulk CD4^+^ T cells edited to overexpress FOXP3 (ectopic [e]Treg) to Treg (CD4^+^CD25^hi^CD127^lo^) and Teff cells (CD4^+^CD25^-^) that were isolated from PBMC and activated and expanded for 12 days (27). As expected, Teff cells had Treg identity scores less than 0 and Treg had scores greater than 0. Of the 4 eTreg samples analyzed, 2 had scores that were above 0 (but lower than the 4 Treg control samples), and the other 2 samples had scores close to 0. Analysis of individual genes in these eTreg samples demonstrated both higher expression of some Teff genes and lower expression of some Treg genes (**Figure 5A**). Compared to SBT Treg-derived cells, eTreg had significantly higher expansion scores (p=0.0002), however their Treg identity score illustrated greater variability and was significantly lower (p=0.03) (**Figure 5B**).

### 3.6 Using the SBT identity and expansion fingerprints to evaluate a clinical-stage Treg cell therapy

**Figure 6:**
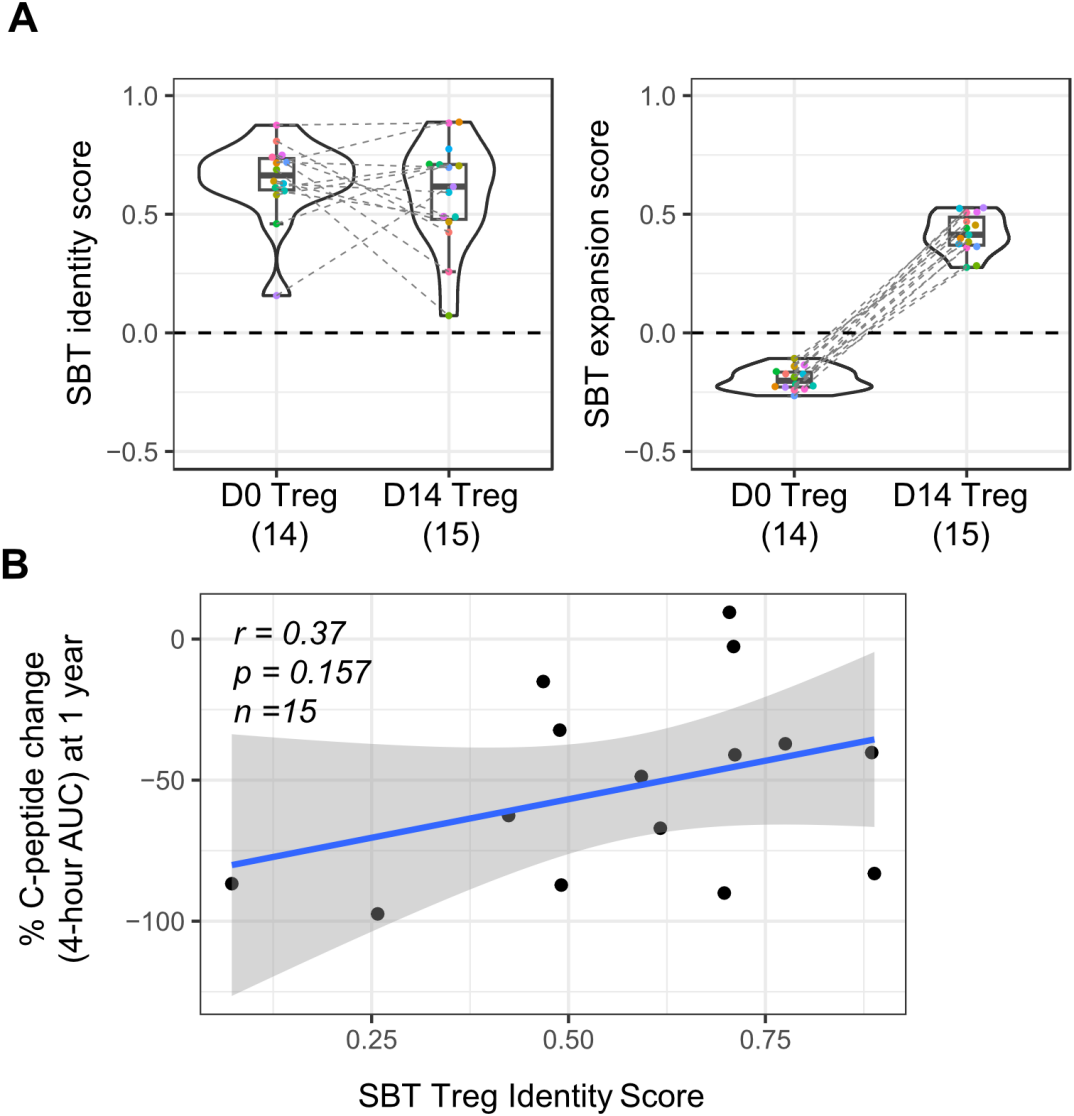
Application of SBT Treg signatures to polyclonal Treg cell therapy drug product used in a phase 2 clinical trial in type 1 diabetes (T1D). **(A)** SBT Treg identity (left) and expansion (right) scores of D0 (baseline/pre-expansion) and D14 (infusion product/post-expansion) Treg from a phase 2 clinical trial in T1D (29). Each color indicates individual participants with dashed lines connecting data from the same participant. **(B)** Correlation between clinical outcome (patient’s percent change in C-peptide AUC at 1 year) and SBT Treg identity scores of D14 Treg infusion product. Blue line represents a linear regression model with the 95% confidence interval shown in shaded gray (Pearson’s r=0.37, Wald test p-value p=0.157).

SBT Treg fingerprints also have the potential to be a tool in the analysis of clinical data. Application of these fingerprints to Treg cell therapy products, either at baseline or pre-infusion, may reveal characteristics predictive of efficacy. To test this hypothesis, we applied the SBT Treg identity fingerprint to published results of a study examining the use of expanded polyclonal Treg for the treatment of T1D (29). Gene expression data was available from baseline (D0) and infusion product (D14) Treg from 14 participants in this phase 2 clinical trial. Application of the SBT Treg identity fingerprint to these samples showed that both baseline and infusion product samples had positive identity scores, suggesting that the starting material for the drug product had an appropriate Treg fingerprint and that the expansion process did not affect the identity of these cells. As shown in **Figure 6A**, identity scores at D14 varied across donors, with some increasing and others decreasing compared to D0, while expansion scores consistently increased at D14.

Though the primary intention of this analysis was to determine if the SBT Treg identity and expansion fingerprints could be successfully applied to D0 Treg and corresponding D14 Treg cell therapy infusion product in this trial, we did further analysis to determine if there was any correlation between the Treg identity score of D14 Treg and a clinical readout from the trial (change in C-peptide percentage AUC after 1 year). While there was a trend in patients who received D14 Treg that had higher Treg identity scores having a smaller decrease in C-peptide 4-hour AUC values 1 year after treatment (correlating with higher insulin production and thus better outcome), significance was not reached (r=0.37; p=0.157) (**Figure 6B**); however, this study was limited by the number of samples available and skewed gene expression distribution because of overall lower sequencing depth compared to internally derived Treg (**Supplementary Figure S3**).

## 4 Discussion

Treg cell therapies have the potential to radically change treatment paradigms for patients with autoimmune diseases. Rather than treating the rampant proinflammatory response with drugs that cause broad immunosuppression in patients, or with drugs that target only one specific aspect of the immune response, bolstering the body’s natural immunosuppressive response by infusion with expanded Treg is an attractive strategy to restore immune homeostasis. As with all autologous cellular therapies, those derived from Treg require careful characterization to ensure that the final product consists of a population of cells that have an appropriate cellular identity and corresponding function. Unlike Teff cell therapies, however, Treg present some additional challenges in this respect. For example, FOXP3, a key transcription factor used to identify Tregs, is expressed only intracellularly, making it unusable for selecting viable cells, and it can also be transiently expressed in activated Teff cells. Analysis of the levels of demethylation of the Treg-specific demethylated region (TSDR) located in the FOXP3 gene can be used to distinguish between Treg (highly demethylated) and Teff (low or no demethylation). However, multiple studies together with our own internal data (not shown) indicate there might be a sex bias in the TSDR methylation level and its impact on Treg phenotypes (45–47). More in-depth methods of characterizing Treg are needed to ensure the identity and function of these promising therapies.

The rise of high-throughput “omics” technologies, including transcriptomics, epigenomics, and proteomics, has revolutionized cellular characterization, enabling unprecedented depth in defining cell states and functions, also known as “fingerprints.” Many published molecular fingerprints have been developed using the gene expression of resting cells, and while it has been demonstrated that these fingerprints can be highly specific in their ability to identify cells of interest, they may not be applicable in cases such as cellular therapies, where cells are stimulated, expanded, and often transduced prior to infusion into patients. To address these gaps, we developed transcriptional fingerprints that can be used to analyze transduced Treg cell therapies using an algorithm that employs bi-directional signatures which define “favorable,” or positive, gene expression signatures and “unfavorable,” or negative, signatures. Calculating a final score by subtracting the negative score from the positive provides a more nuanced measure of the quality of Treg cell therapies.

Sonoma Biotherapeutics has formulated 2 Treg fingerprints: an identity fingerprint to differentiate between Treg and Teff regardless of their expansion state, and an expansion fingerprint to distinguish cells (Treg or Teff) that have undergone *in vitro* expansion. When applied to a large internal data set of D0 (pre-expansion) and D14 (post-expansion) Tregs and Teffs, both performed well, assigning positive scores to cells with favorable characteristics and negative scores to those with unfavorable ones. For the expansion fingerprint, both Treg and Teff had scores below 0 at D0, and above 0 at D14, despite the fingerprint being trained solely on Tregs. Applying the Treg identity fingerprint, Treg at both time points had scores above 0, while Teff had scores below 0. Notably, the identity fingerprint outperformed FOXP3 expression alone in distinguishing D14 Teffs from both D0 and D14 Tregs. The performance of the SBT identity fingerprint was also benchmarked against two published signatures: one by Ferraro et al. (18), which misclassified D14 Teffs as Treg-like, and another by Pesenacker et al. (19), which did not, likely due to its inclusion of activated Tregs in the training set, yielding a signature more comparable to the SBT Treg identity fingerprint.

Together with the fingerprint’s performance across multiple Treg and Teff datasets, we also assessed the impact of minor processing variation to determine its suitability for use in practical workflows. Previous studies have shown that short-term processing delays only minimally affect gene expression profiles (48). Identity scores remained consistent following a 24-hour post-thaw rest period, supporting their robustness under real-world handling conditions. In contrast, expansion scores showed greater variability during this interval, though there was no significant overall change. This variability likely reflects donor-specific differences in proliferative state at the end of culture. These findings suggest that while the identity fingerprint may be reliably applied across varied settings, the expansion fingerprint may benefit from standardized processing to ensure consistent interpretation.

Aside from being used to confirm the identity and expansion states of SBT Treg, we demonstrated that our Treg fingerprints could be applied to other data sets to glean additional insights that may not be captured in traditional analyses. We demonstrated one potential use by comparing Treg that expressed a CAR which demonstrates high levels of tonic signaling (leading to destabilization of FOXP3 expression and a decrease in immunosuppressive function) to D0 and D14 Treg and Teff. The Treg identity scores of destabilized Treg were lower than those of D0 and D14 Treg, and closer to scores of Teff, indicating that these chronically stimulated cells lose some aspects of the Treg phenotype. In autoimmune diseases such as RA, multiple studies have demonstrated that patient-derived Treg show some level of phenotypic abnormalities and dysfunction/destabilization (23, 49). In the case of autologous Treg cell therapies that need to be manufactured using patients’ cells, little is known about the potential impacts of these abnormalities on the final drug product. The SBT Treg fingerprints are sensitive enough to enable a more nuanced view of Treg identity; comparison of the identity scores of final drug product generated from healthy donors or patients with autoimmune diseases might lead to insights on whether the manufacturing process “restores” dysfunctional Treg or findings that could inform changes in manufacturing to overcome these deficiencies.

In another case, we compared the Treg identity scores of CD4^+^ T cells that had forced expression of ectopic FOXP3 (eTreg) with Treg and Teff cells. eTregs exhibited scores that generally fell between those of Teffs and bona fide Tregs. FOXP3 overexpression is one strategy to mitigate the risk of Treg instability, as Tregs exposed to proinflammatory conditions can lose FOXP3 expression and adopt a proinflammatory phenotype, potentially exacerbating autoimmune disease. Although eTregs have demonstrated immunosuppressive activity *in vitro* and, to some extent, in *in vivo* models, their function has not yet been clinically validated. The lower SBT Treg identity scores observed in eTreg suggest that while overexpression of FOXP3 can impart partial regulatory features, these cells still retain aspects of a Teff-like transcriptional profile.

Ultimately, the ideal application of these Treg fingerprints is to correlate them with clinical results, enabling the prediction of patient response and informing next-generation manufacturing and engineering strategies. We demonstrated the applicability of SBT Treg fingerprints to polyclonal Tregs in a T1D clinical trial (29) and observed a promising trend between SBT Treg identity scores in the pre-infusion products and clinical outcome (r=0.37), although this did not reach statistical significance. This analysis was limited by several factors, including a small sample size (n=15), sequencing depth of these samples that was generally lower than internally derived samples, and the absence of a clinically meaningful treatment effect in the T1D patients. Given previous studies in CAR-T therapy have shown gene-expression signatures to be more predictive than conventional phenotyping (50), our observation supports the potential utility of Treg fingerprints as a quantitative biomarker in clinical trials. Future work involving matched transcriptional profiling of pre-infusion products and comprehensive clinical annotation will be critical to further validate the Treg fingerprints as a clinically useful biomarker. If validated, this fingerprint could play a key role in ensuring batch consistency and guiding dose selection in CAR Treg therapy. For example, products with higher Treg fingerprint scores might warrant lower cell doses, whereas lower-scoring lots could indicate the need for higher doses. Additionally, measuring the signature in patient blood or tissue samples at baseline could enable identification of more responsive patients, facilitating patient stratification and enrichment strategies in future clinical trials.

Due to the application of bi-directional signatures and sub-score design, our fingerprints have demonstrated a robust threshold of 0 for discriminating Treg vs. Teff samples, as well as expanded vs. non-expanded samples. However, defining thresholds for ‘high-quality’ vs. ‘suboptimal’ Treg products that strongly correlate with clinical outcomes remains speculative at this stage and will require robust clinical data. Building upon the fingerprints’ existing discriminatory power, we can propose potential criteria based on our findings and the existing literature. For the Treg identity score, in addition to a possible threshold of 0, a more stringent threshold based on the distribution of scores in our study could be investigated; for example, products falling below the 25th percentile could be considered ‘suboptimal’, warranting further investigation into the manufacturing process. Regarding expansion scores, which reflect the cells’ proliferative capacity, a threshold could be set based on a minimum fold-increase during expansion where products falling below this threshold might be deemed less likely to respond to stimuli and persist *in vivo*. Ultimately, these thresholds would need to be refined and validated in larger cohorts with well-annotated clinical data, correlating product characteristics with patient outcomes.

Although the identity and expansion state of Treg cell therapies are important to fully characterize as part of the drug development process, these fingerprints may not fully capture perhaps the most important aspect of a successful cell therapy: potency. Treg can exert immunosuppressive function in multiple ways, including secretion of suppressive cytokines (IL-10, TGF-β, IL-35), metabolic disruption of effector T cells (high IL-2 consumption, adenosine production, tryptophan depletion), and direct cell-to-cell contact (CTLA-4, LAG3), in a sense acting as a cellular “polypharmacy” to dampen inflammation (51). Current potency assays for Treg cell therapies primarily assess their ability to suppress T cells, however these assays can vary in format and endpoint and primarily reflect IL-2 consumption and may not capture other key suppressive mechanisms (52). Additionally, Treg can affect other cell types, including antigen presenting cells (53, 54), which is not captured in T cell suppression assays. As such, direct measurement of the potency of each of these functions is difficult to analyze for each patient-derived drug product. Despite these limitations and the small number of samples, the Treg identity fingerprint showed a promising correlation with *in vitro* suppressive potential, supporting the potential utility of this approach and motivating further investigation. In future studies, mechanism-specific transcriptional signatures could be developed to capture gene expression associated with distinct immunosuppressive pathways. These could complement the identity and expansion scores to provide a more holistic view of Treg cell therapy products prior to infusion.

Although our Treg fingerprints have been successfully applied across multiple applications, demonstrating their ability to distinguish between different cell states and conditions, several limitations should be considered. First, our analysis assessed immune cell subsets using subset-specific gene expression signatures at the bulk level. While Treg isolation protocols yield highly purified populations of cells prior to expansion, 100% purity is not guaranteed. Our fingerprints, developed using bulk gene expression analysis, are not optimized to detect rare contaminating cell subsets. However, the design of the fingerprint combining “favorable” and “unfavorable” cell subsets using a weighted scheme allows further optimization based on the objective, such as detecting minimally allowable “unfavorable” cell subsets. Second, the selection of genes included in the Treg fingerprints is inherently a limitation for any gene signature approaches, as alternative gene sets could potentially yield different results. While we employed ssGSEA, a non-parametric method, to mitigate potential batch effects across datasets, we cannot entirely rule out the influence of subtle, uncorrected variations such as differences in sequencing depth or variations in RNA input amount. Finally, while we have identified promising Treg fingerprints, further validation in larger, independent cohorts is needed to confirm their robustness and clinical utility.

In summary, we have developed robust and predictive Treg identity and expansion fingerprint algorithms that integrate the gene expression signatures of both favorable and unfavorable cell characteristics in Treg cell therapy drug products. This ssGSEA-based scoring system offers a straightforward and reproducible framework for assessing cellular identity and expansion state, and can be adapted for broader applications in other cell therapy products or immune cell types. Ongoing efforts are focused on further validating these fingerprints through targeted gene expression assays, such as NanoString, to confirm their robustness and reproducibility beyond bulk RNA-seq. In addition, it would be valuable to conduct epigenomic profiling, including chromatin accessibility and DNA methylation analyses, to determine whether stable regulatory features support these transcriptional fingerprints. Beyond validation of existing fingerprints, future research is needed to define additional fingerprints associated with distinct functional pathways, enabling a more nuanced characterization of CAR-Tregs that may act through multiple mechanisms. Importantly, while these fingerprint scores provided a valuable tool for assessing product quality, consistency, and therapeutic potential, they should be considered as part of a comprehensive evaluation strategy that incorporates multi-omics approaches and functional assays that can provide a more holistic understanding of these complex drug products. As the field of cell therapy continues to advance, our fingerprinting approach lays a foundation for improving product characterization, ensuring product safety, and ultimately enhancing clinical outcomes.

## Data Availability

The SBT cell therapy product RNA-seq datasets presented in this article are not deposited in an open access data repository because they contain Sonoma Biotherapeutics proprietary information on our clinical product. Requests to access the datasets should be directed to the corresponding author.
