# Supplementary material for: Transcriptional fingerprinting of regulatory T cells: ensuring quality in cell therapy applications
Source: Front Immunol. 2025 Jun 16;16:1602172. doi: 10.3389/fimmu.2025.1602172 (PMC12206644; doi:10.3389/fimmu.2025.1602172)
Supplement: Supplementary file 1 [file DataSheet1.docx]

Supplementary Material

# Supplementary Figures and Tables

## Supplementary Figure S1.


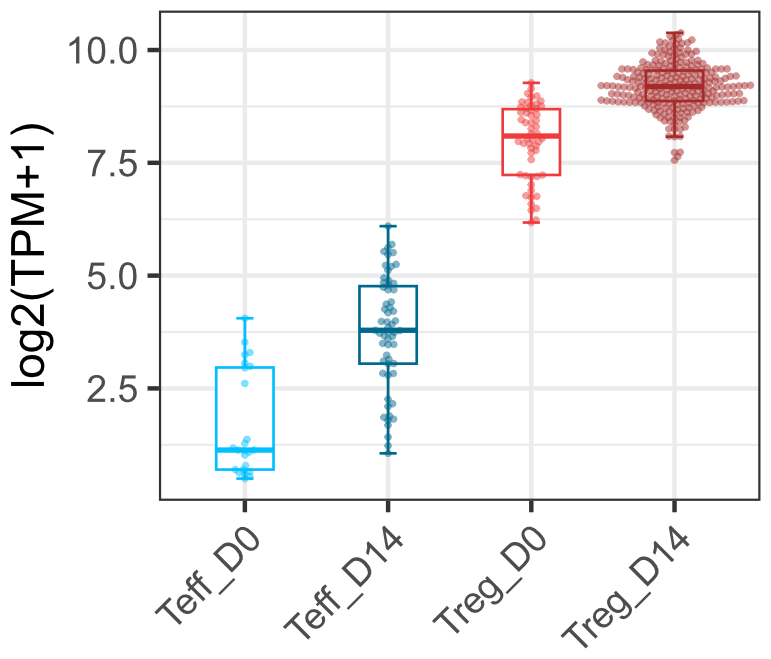


**Supplementary Figure S1.** Expression of FOXP3 RNA in D0 and D14 Teff and Treg as measured by bulk RNAseq from all internally generated datasets.

## Supplementary Figure S2.


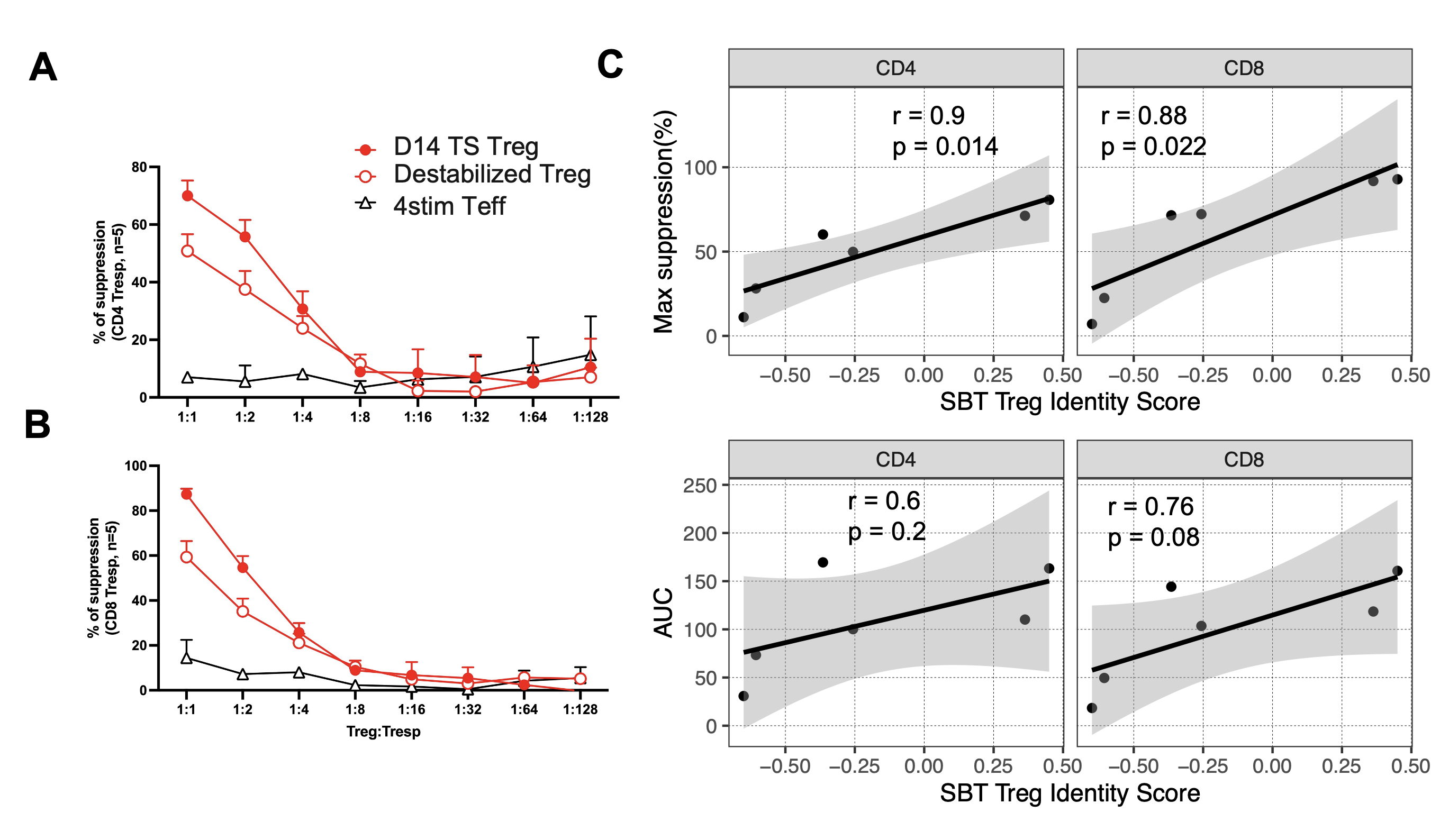


**Supplementary Figure S2. Destabilized Tregs exhibit reduced suppression capacity that correlates with SBT Treg identity score.** Suppression of (A) CD4⁺ and (B) CD8⁺ T cell proliferation by Day 14 Tregs expressing high-tonic signaling CAR (tsTreg; red closed circles), destabilized Tregs (red open circles), and 4stim Teff (black open triangles) (n=5). (C) Correlation between SBT Treg identity score and suppression capacity, quantified by maximum percentage suppression (top) and area under the curve (AUC) (1) across Treg:Tresp ratios from 1:1 to 1:128 (bottom). Panels A and B represent data from two donors with matched suppression and identity scores. Black line represents a linear regression model with the 95% confidence interval shown in shaded gray. Pearson correlation coefficients (r) and p-values are shown.

## Supplementary Figure S3.


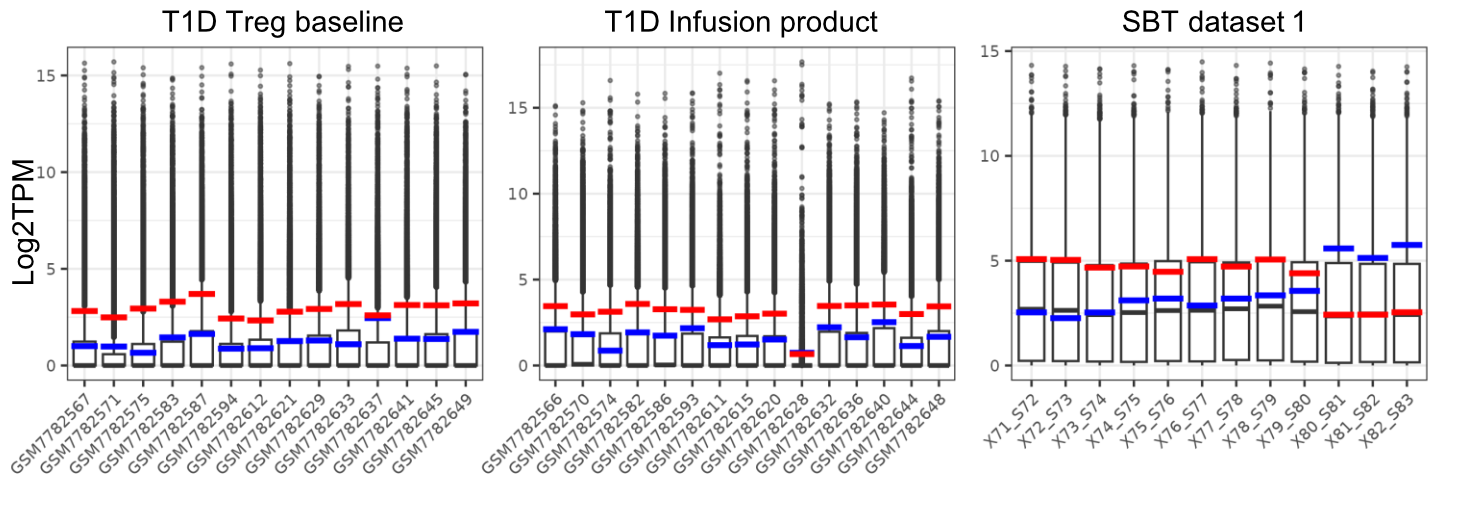


**Supplementary Figure S3.** Box plots of log2TPM values for all genes, highlighting the median expression of Treg (red) and Teff (blue) signature genes across 3 sample groups: baseline (D0; left) and infusion product (D14; middle) Treg generated from T1D patients (2) and an internal Sonoma Biotherapeutics (SBT) dataset (right).

## Supplementary Table S1. List of Datasets Used in SBT Treg Fingerprint Discovery, Validation, and Application

| **Dataset_name** | **Source** | **Use** | **D0**  **Teff** | **D0**  **Treg** | **D14 Teff** | **D14 Treg** | **Destab. Treg** | **4 Stim Teff** |
| --- | --- | --- | --- | --- | --- | --- | --- | --- |
| SBT dataset 1 | Internal | Discovery (Identity) | 0 | 0 | 3 | 9 | 0 | 0 |
| SBT dataset 2 | Internal | Discovery (Identity & Expansion) | 8 | 8 | 18 | 36 | 0 | 0 |
| SBT dataset 3 | Internal | Discovery (Expansion) | 0 | 22 | 0 | 12 | 0 | 0 |
| SBT dataset 4 | Internal | Validation | 0 | 0 | 0 | 8 | 0 | 0 |
| SBT dataset 5 | Internal | Validation | 0 | 0 | 0 | 56 | 0 | 0 |
| SBT dataset 6 | Internal | Validation | 0 | 0 | 0 | 24 | 72 | 0 |
| SBT dataset 7 | Internal | Validation | 16 | 18 | 9 | 35 | 0 | 0 |
| SBT dataset 8 | Internal | Validation & Application | 0 | 3 | 3 | 6 | 0 | 3 |
| Mensink et al. 2024 (3) | External | Validation | 0 | 0 | 10 | 10 | 0 | 0 |
| SBT dataset 9 | Internal | Validation | 0 | 0 | 4 | 4 | 16 | 0 |
| SBT dataset 10 | Internal | Validation | 0 | 0 | 0 | 3 | 0 | 0 |
| SBT dataset 11 | Internal | Validation | 0 | 3 | 0 | 13 | 11 | 0 |
| SBT dataset 12 | Internal | Validation | 0 | 0 | 12 | 36 | 0 | 0 |
| Honaker et al. 2020 (4) | External | Application | 0 | 0 | 4 | 8 | 0 | 0 |
| Bender et al. 2024 (2) | External | Application | 0 | 14 | 0 | 15 | 0 | 0 |

Abbreviations: Destab. = destabilized; SBT = Sonoma Biotherapeutics; Stim = stimulation; Teff = effector T cells; Treg = regulatory T cells

## Supplementary Table S2. Genes Included in SBT Treg Identity and Expansion Fingerprints.

See Excel file.

## Supplementary Table S3. Comparison of Diagnostic Performance Metrics Across SBT and Published Fingerprints

| **Metric** | **SBT Identity Fingerprint** | **Ferraro et al. 2014 (5)** | **Pesenacker et al. 2016 (6)** |
| --- | --- | --- | --- |
| Accuracy | 100% | 86% | 99% |
| Sensitivity | 100% | 100% | 100% |
| Specificity | 100% | 34% | 98% |
| PPV | 100% | 85% | 99% |
| NPV | 100% | 100% | 100% |

Abbreviations: NPV = negative predictive value; PPV = positive predictive value; SBT = Sonoma Biotherapeutics

**References**

1. Akimova T, Levine MH, Beier UH, Hancock WW. Standardization, Evaluation, and Area-under-Curve Analysis of Human and Murine Treg Suppressive Function. *Methods Mol Biol* (2016) 1371:43-78. doi: 10.1007/978-1-4939-3139-2_4.

2. Bender C, Wiedeman AE, Hu A, Ylescupidez A, Sietsema WK, Herold KC, et al. A Phase 2 Randomized Trial with Autologous Polyclonal Expanded Regulatory T Cells in Children with New-Onset Type 1 Diabetes. *Sci Transl Med* (2024) 16(746):eadn2404. Epub 20240508. doi: 10.1126/scitranslmed.adn2404.

3. Mensink M, Verleng LJ, Schrama E, Janssen GM, Tjokrodirijo RT, van Veelen PA, et al. Tregs from Human Blood Differentiate into Nonlymphoid Tissue-Resident Effector Cells Upon Tnfr2 Costimulation. *JCI Insight* (2024) 9(5). Epub 20240308. doi: 10.1172/jci.insight.172942.

4. Honaker Y, Hubbard N, Xiang Y, Fisher L, Hagin D, Sommer K, et al. Gene Editing to Induce Foxp3 Expression in Human Cd4(+) T Cells Leads to a Stable Regulatory Phenotype and Function. *Sci Transl Med* (2020) 12(546). doi: 10.1126/scitranslmed.aay6422.

5. Ferraro A, D'Alise AM, Raj T, Asinovski N, Phillips R, Ergun A, et al. Interindividual Variation in Human T Regulatory Cells. *Proc Natl Acad Sci U S A* (2014) 111(12):E1111-20. Epub 20140307. doi: 10.1073/pnas.1401343111.

6. Pesenacker AM, Wang AY, Singh A, Gillies J, Kim Y, Piccirillo CA, et al. A Regulatory T-Cell Gene Signature Is a Specific and Sensitive Biomarker to Identify Children with New-Onset Type 1 Diabetes. *Diabetes* (2016) 65(4):1031-9. Epub 20160119. doi: 10.2337/db15-0572.
